# The impact of social violence on HIV risk for women in Colombia: A concurrent mixed methods study

**DOI:** 10.1371/journal.pgph.0001571

**Published:** 2023-02-24

**Authors:** Catalina Correa-Salazar, Ariela Braverman-Bronstein, Usama Bilal, Ali K. Groves, Kathleen R. Page, Joseph J. Amon, Alejandra Vera, Laura Ballesteros, Ana Martínez-Donate

**Affiliations:** 1 Department of Community Health and Prevention, Drexel University, Philadelphia, Pennsylvania, United States of America; 2 Epidemiology and Biostatistics Department, Drexel University, Philadelphia, Pennsylvania, United States of America; 3 Johns Hopkins School of Medicine, Johns Hopkins University, Baltimore, Maryland, United States of America; 4 Corporación Mujer Denuncia y Muévete NGO, Cúcuta, Colombia; UNAM: Universidad Nacional Autonoma de Mexico, MEXICO

## Abstract

Gender, violence, and migration structurally impact health. The Venezuelan humanitarian crisis comprises the largest transnational migration in the history of the Americas. Colombia, a post-conflict country, is the primary recipient of Venezuelans. The Colombian context imposes high levels of violence on women across migration phases. There is little information on the relationship between violence and HIV risk in the region and how it impacts these groups. Evidence on how to approach the HIV response related to Venezuela’s humanitarian crisis is lacking. Our study seeks to 1) understand how violence is associated with newly reported HIV/AIDS case rates for women in Colombian municipalities; and 2) describe how social violence impacts HIV risk, treatment, and prevention for Venezuelan migrant and refugee women undergoing transnational migration and resettlement in Colombia. We conducted a concurrent mixed-methods design. We used negative binomial models to explore associations between social violence proxied by Homicide Rates (HR) at the municipality level (n = 84). The also conducted 54 semi-structured interviews with Venezuelan migrant and refugee women and key informants in two Colombian cities to expand and describe contextual vulnerabilities to HIV risk, prevention and care related to violence. We found that newly reported HIV cases in women were 25% higher for every increase of 18 homicides per 100,000, after adjusting for covariates. Upon resettlement, participants cited armed actors’ control, lack of government accountability, gender-based violence and stigmatization of HIV as sources of increased HIV risk for VMRW. These factors impose barriers to testing, treatment and care. Social violence in Colombian municipalities is associated with an increase in newly reported HIV/AIDS case rates in women. Violence hinders Venezuelan migrant and refugee women’s access and engagement in available HIV prevention and treatment interventions.

## 1. Introduction

Structural determinants of health involve interconnected institutions whose linkages are historically rooted and culturally reinforced through stigmatizing beliefs, values and distribution of resources [1], impacting individual and community health. These factors are external to the individual and are produced through a dynamic interplay of macrostructural factors (i.e., social, economic and health policies, laws, mobility and migration, sociopolitical transitions, stigma, cultural norms on gender and sexuality); community organization (e.g., values, participation, peer structures of care); and the physical, social, economic, and policy features of environments [2]. hat contributes to oppression through gender, ethnic, and class-based discrimination [3].

We conceptualize gender [4] and migration [5, 6] as structural factors impacting women’s and girls’ health crossing transnational borders through structural and systematic violence enforced against them as individuals immersed in structural determinants of health. Violence is built into the structure and shows up as unequal power and consequently as unequal life chances [7] for migrant and refugee women and girls. These phenomena respond to historical, systemic, and political forces beyond social determinants of health (i.e. neighborhood characteristics, housing, access to care, safety) [8] and are ways in which societies foster and reproduce discrimination, segregation, unequal distribution of resources and physically and psychologically injure minorities [9]. Altogether, gender, migration and violence affect the risk of experiencing adverse health outcomes [1, 8].

Migrants are one of the most vulnerable groups to HIV infection, consequences and risks [10] given higher risk of HIV acquisition and onward transmission across migration phases (pre-departure, departure, transit, arrival) [10, 11]. Risk of HIV infection across these phases is highly gendered [11], linking geographically separate epidemics and intensifying transmission through violence and riskier sexual behaviors [12]. Worldwide, the risk of suffering any kind of violence for women living with HIV (WLHIV) is 2 to 16 times higher when compared to women not living with HIV [13].

Being a migrant [14], a woman (cis and trans) and living with HIV intensifies stigma through associations with power, disease, and othering [15, 16]. Stigma is the social process of labeling, stereotyping and rejecting human difference to enforce control [17]. Stigma, as embedded into structural determinants of health, is a violent practice enacted through behaviors, beliefs and ideologies. Stigma is intersectional. Thus, socially stigmatized characteristics can add and/or to the end result of disparities [18, 19]. We draw from public health research in this study to analyze how multiple interrelated features interact to produce health outcomes [20] for certain populations, in this case: being a woman, a migrant and be perceived to live with HIV, in addition to other features like gender identity and sexual orientation, which further reinforce discrimination.

The Venezuelan humanitarian crisis comprises the largest transnational migration in the history of the Western Hemisphere [21]. Venezuela’s underfunding of the healthcare system has left its population unprotected and untreated for major health conditions [22] including HIV and AIDS (for further context see elsewhere) [23, 24]. Venezuela is one of few middle-income countries where people living with HIV (PLHIV) have been forced to interrupt their treatment [22]. Colombia is the primary recipient of Venezuelan migrants and refugees [25], who mainly relocate to large urban centers [26]. The Colombian post-armed conflict context imposes extremely high levels of violence on women [27].

As of 2018, there were around 2.1 million people living with HIV in Latin America and the Caribbean (LAC), of which nearly half were women [28], most of reproductive age (15–49). In LAC, the HIV epidemic has been historically concentrated in urban areas and among key populations (gay men, sex workers, intravenous drug users and transgender women) [29] and is impacted by gender inequalities [13]. In Colombia, HIV/AIDS incidence has increased by 10% among adults (ages 18–49) between 2014–2017 in urban populations receiving heavy migration flows from Venezuela [30, 31]^.^ HIV mortality in women has increased by 19.5% for the same time period [29], temporally coinciding with the start of the increase in migration flows [32]. Late-access to diagnosis has been related to migration status, stigma, discrimination, gender-based violence (GBV) and gender inequality [29]. In LAC, the HIV epidemic is influenced by sociopolitical and economic factors [33] like poverty, job security, educational attainment, living conditions and poor access to healthcare services [34], all of which are also associated to increased homicide rates (HR) [35].

### 1.1. Homicide rates (HR) and HIV risk for migrant/refugee women in Colombia

LAC is one of the most unequal and urbanized regions worldwide [36]. Potentially as a result of these patterns, LAC has the highest HR in the world [37], a major health problem [38] especially in urban areas in Colombia [39]. Homicide, defined as intentional death that one person causes on another, is considered a key indicator of levels of violence (including interpersonal violence, injuries, presence of organized crime and lack of governance) [40] in a specific area, country or region [38]. As a result, HR are considered a reliable measure of social violence (which includes more than two people and occurs in a community and/or national context) [37] and have been related in post-conflict countries [40] to detrimental effects on city growth [39], development, democratic consolidation and human rights protections [40]. HR are strongly correlated to poverty [39], inequality, impunity, corruption, and organized crime [37]. Given strong evidence supporting HR as a proxy for social violence in LAC, we use the terms interchangeably [40].

Colombia is considered a high-violence country [35, 39, 41], with a HR of 25 per 100,000 people, three times the global rate [42]. HR in Colombia have been related to the prevalence of organized crime [37], drug trafficking and criminal economies [43], size of urban population, lack of schooling, poverty [41], impunity, institutional weakness [43] and the war on drugs [44]. In Colombia, most homicides occur in urban areas [39]. although violence encompasses most of the country [37] and increases in relation to the presence of armed groups [43].

Illegal armed groups controlling borderlands and urban territories in Colombia represent a myriad of dangers for women across migration phases [27, 45]. Gender inequality is strongly associated with increased HR for both men and women [46]. Colombia’s high femicide rate, defined as the homicide of a woman on account of her gender identity, and high rates of sexual violence against women makes it a violent country towards women [47]. This violence is especially dire against Venezuelan migrant and refugee women (VMRW), making them a target of selected killings [48]. VMRW make up half of the total Venezuelan migrant influx and re-settled population [25] and are a key vulnerable group with significant health needs related to HIV/AIDS care and treatment [22, 30, 31]. maternal and child health, sexual and reproductive health [49, 50], infectious [51] and chronic conditions and access to health services [23, 24]. For VMRW access barriers related to migration status and stigma hinders HIV testing, prevention and treatment [29]. In Colombia, armed actors selectively kill, threaten and displace PLHIV, especially WLHIV, as a “social cleansing” strategy [52].

Health is a right in Colombia under the Constitution. In response to the increasing incoming Venezuelan migration flows, the Ministry of Health established that migrants/refugees (including those with irregular migration status) are entitled to emergency care (including prenatal care and some reproductive health services) and public health actions [53]. Health policies for international migrants, refugees, and asylum seekers in Colombia are based in a human rights framework and immigrants are entitled to health care with limitations depending on migration status (i.e. legal versus unauthorized) [54]. The Temporary Protection Law, passed in response to the crisis, has been presented as a long term solution that promotes integration and inclusion in social support systems (including healthcare and education, among others) through migratory permits for Venezuelans [55]. However, this law does not comprise a holistic set of migratory policies that cover specific aspects of health, barriers to healthcare or access to justice. Colombia has yet to adopt a set of comprehensive health policies that address the migrant/refugee population’s needs and tackle specific barriers to the right to health. To effectively respond to these needs, local programmatic responses that integrate government and non-government actors are paramount [56].

There is little information on the relationship between migration, violence, and HIV risk in LAC [37], particularly on how to address HIV in migrant populations and WLHIV [33]. There is a need for evidence to understand how violence in Colombia [43] impacts HIV risk and mortality for women in general and migrant women in particular, considering high levels of gender inequality, increasing HR -especially among women migrants- and HIV stigma. Evidence in the region on how to approach the HIV response related to Venezuela’s humanitarian crisis is lacking [57]. Given that migration has been linked to structural barriers for HIV care, testing, adherence to treatment, and health systems barriers in Colombia and LAC [58], particularly for migrants and women in armed-conflict and humanitarian contexts [59], research addressing these issues is pivotal. In this study, we aim to 1) understand how violence is associated with newly reported HIV/AIDS case rates for women in Colombian municipalities following the hypothesis that at the municipal level, higher HR are associated with higher HIV rates; and 2) describe how social violence impacts HIV risk, treatment, and prevention specifically for VMRW undergoing transnational migration and resettlement in two Colombian cities.

## 2. Methods

**Fig 1:**
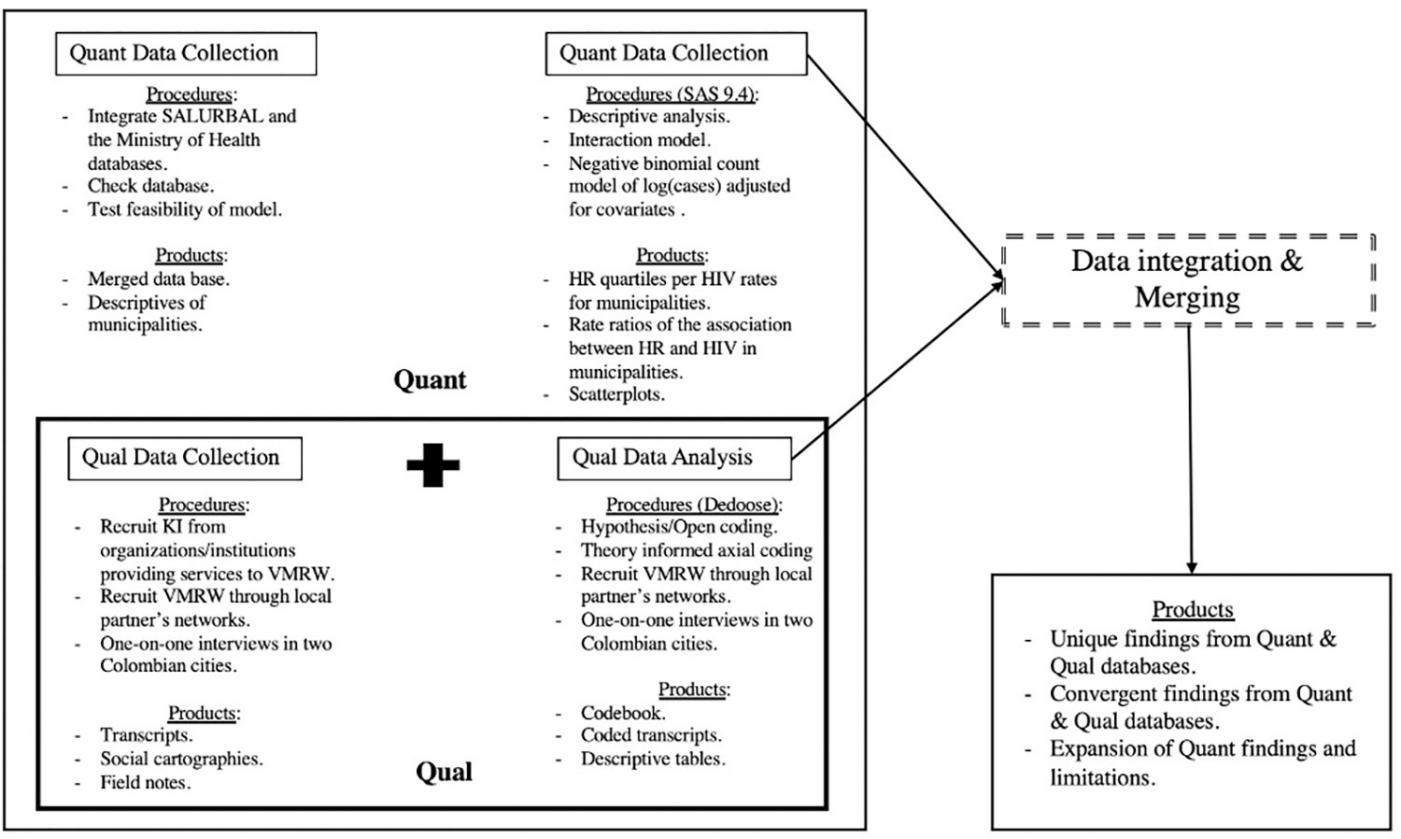
Schematic of concurrent (Quant + Qual) design [44].

We used a mixed method concurrent (Quant + Qual) design (see Fig 1) [60, 61] to understand how violence modifies HIV risk in newly reported HIV/AIDS cases for foreign-born and Colombian women in Colombian municipalities. The concurrent design allowed us to collect different forms of data simultaneously and iteratively, analyze the data, and merge research findings to understand and expand quantitative results (see our convergence matrix in integration of results) [61, 62]. The iterative approach allowed us to shift the focus to respond to arising gaps in quantitative data [62]. Despite the latter was part of the 2018 census, we collected it from the Ministry of Health’s databases in 2021 and all analyses were conducted that year.

### 2.1 Quantitative component: Measures and statistical analysis

For the quantitative ecological part, we used data on the 84 urban Colombian municipalities that are part of the Urban Health in Latin America project (SALURBAL), a multisite collaborative project that gathers data related to health, environment, and social indicators at the individual, neighborhood, and city level for urban centers in Latin America [63]. The SALURBAL project has compiled and harmonized health, social and physical environment data on all cities with a population above 100,000 in 11 Latin American countries (including Colombia) [64]. Cities of ≥100,000 people were identified by combining information from the Atlas of Urban Expansion, census-based population data on administratively defined cities in each country and inspection of built-up areas on satellite maps [63, 64] Using this approach, 35 cities in Colombia were identified and operationalized as clusters of the smallest administrative units (municipalities) for which disaggregated vital statistics data were available. This analysis yielded 84 municipalities pertaining to 35 cities, which comprise altogether 61.6% of the Colombian population [64].

We collected publicly available information on newly reported HIV/AIDS cases by country of origin (Colombia and foreign-born) among women aged 18 or above from the Colombian Ministry of Health and Social Protection. These numbers refer to cisgender women only, there is no data for transgender women. We operationalized incident cases as newly reported/detected cases in Colombia for 2018, understanding it is nearly impossible to assess when/where migrants/refugees were infected and if their case was reported previously in another country. To calculate newly reported HIV/AIDS case rates, we used population denominators obtained from the 2018 census and proxied social violence by the number of homicides (HR) per 100,000. Data was collected and analyzed in 2021. Covariates are indicated in Table 1.

**Table 1:** Main outcome & covariates.

	Variable	Definition
Outcome	HIV/AIDS newly reported cases in women by nativity	Newly reported cases of HIV/AIDS by municipality and place of origin (foreign-born vs Colombia), divided over the number of people living in the municipality by place of origin according to 2018 census.
Exposures	Social Violence	Homicide rate per 100,000 people. Homicide rate is a common and reliable proxy to estimate social violence.
Covariates	Poverty and general living conditions score^	% households with overcrowding, % of households with piped water inside the dwelling, % of adolescents 15–17 years that go to school.
	Population education**	% of the population 25 years old or more with complete high school education or more
	Population size	Number of people living in each municipality.
	Population growth	% change in the population over the period 2013–2018.
	Service provision score**	% of households with water from a public network and % of households connected to a public sewage network
	% migrant population	% of migrant population re-settled in Colombian municipalities
	% of total female migrant population	% of total female migrant population re-settled in Colombian municipalities.

^These scores were created using a principal component analysis with the SALURBAL data for 2005.

**These scores were created using a principal component analysis with the SALURBAL data for 2018.

Covariates are composite indicators characterizing the social and economic environment: living conditions (including indicators of overcrowding), population education (including high school attendance) and service provision (including sanitation). These scores were developed by the SALURBAL project team using principal component analysis for previous studies and have been found to be associated with several health outcomes. Higher scores indicate better socioeconomic environment.

We estimated descriptive statistics of the outcomes and covariates for 84 Colombian municipalities in each of the four quartiles of homicide rates (HR). To explore associations between social violence, proxied by HR obtained from vital registration, and newly reported HIV case rates obtained from the Colombian Ministry of Health, we fitted negative binomial models with HIV/AIDS newly reported cases in women as the outcomes. We fitted unadjusted and adjusted models including covariates in Table 1. We also fitted a model including an interaction term for the population of foreign-born and HR quartiles to test the hypothesis that at the municipal level, higher HR and larger foreign-born populations are associated with higher HIV rates. Finally, we conducted a pairwise comparison using Tukey test, which yielded no significant differences in HR between quartiles

### 2.2 Qualitative component: Interviews and analysis

**Fig 2:**
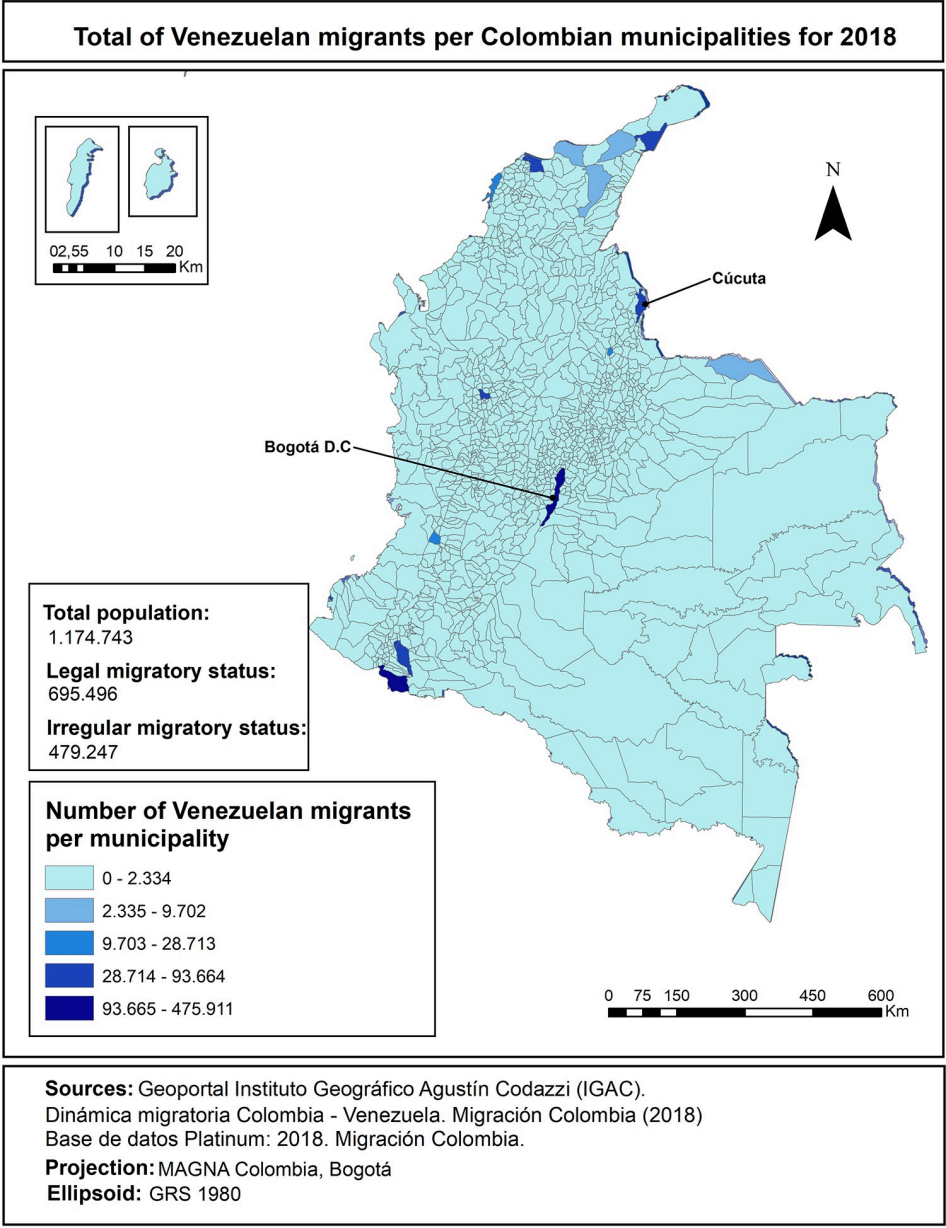
Venezuelan migrants per Colombian municipalities for 2018.

We conducted semi-structured interviews (n = 54) in the two Colombian cities with the highest number of re-settled migrants (see Fig 2) [26]. Bogotá, the capital and historically a receptor of displaced populations [26], is where most migrants/refugees relocate given availability of health and social resources. Cúcuta is the main border city and where most incoming women from Venezuela seek healthcare, medications and treatment [49, 65]. Qualitative work was co-conducted by a local partner organization and community educators, following a participatory-informed framework [66]. The partnership was built upon a three-year collaboration.

#### 2.2.1 VMRW sampling and recruitment

We followed a *non-probabilistic purposive snowball and convenience sampling* model to conduct n = 54 interviews intended to achieve qualitative diversity principles [67, 68]. The PI of the study and the local partner were responsible for conducting all interviews in Spanish. VMRW (N = 30 / n = 15 per study site) were sampled for diversity in HIV status, migratory status, and transactional sex experiences between January and July 2021. Participants were recruited through our local partner and were given an incentive of $15 USD, a reasonable amount to compensate their time per Colombian standards. Participants were given incentives to acknowledge their time investment in the research as contributors according to our PAR framework [69]. All participants provided verbal consent to participate, including underaged emancipated adolescents. The IRB specifically waived the need for parental/guardian consent for minors. All VMRW were between 14–49 years of age (reproductive age aligning with group most affected by HR, largest group of the migrant/refugee population, and group with the highest growing rate of HIV/AIDS) [31]; living in Venezuela before 2013 (migrated to Colombia within the last 7 years to capture largest migration waves and census year 2018); and re-settled in Bogotá or Cúcuta after terrestrial transnational migration (having been exposed to border crossing and transit) [6].

#### 2.2.2 VMRW sample

Main demographics of VMRW sampled for the qualitative phase represent mean age for women was 28 years old and majority of the sample migrated between 2017–2018 due to the economic crisis in Venezuela (50%) and health reasons (20%). Almost half of cisgender women engaged in sex work and all the transgender women. Around a third had other forms of informal work (27%), which means they did not have access to social benefits, or to the public health network (including insurance) through work. Over half (53%) used an illegal crossing to enter Colombia and the majority did not have any type of migratory permit (90%). Most women have children (67%), have been a teenage mother (53%), have suffered the abandonment of at least one parent (67%), and considered their family exposed to violence after re-settlement (67%).

#### 2.2.3 Key informants sampling and recruitment

Key informants (KI) (n = 12 per study site, n = 24 total) were sampled through referral networks. All KI were sampled from organizations providing direct services to migrants. 25% of KI work in government institutions, 4% in academia, 4% in healthcare settings and 22% in civil society organizations. KI were 75% female and almost half (46%) worked in international non-profit organizations.

#### 2.2.4 Interview data analyses

All interviews were audio recorded and transcribed verbatim by research assistants. Transcriptions were reviewed by the PI for quality purposes. All interviews were coded in Spanish by a team composed of the PI, a member of the local partner organization and three local research assistants. Interviews were cleaned, summarized, and anonymized for coding. Initial codes were discussed and reconciled by all team members. Codes were iteratively modified, applied, and reconciled by team members and once all transcripts were coded [70], axial themes, organized in main findings, were structured as results using memos, emergent themes, important quotes, and Dedoose 9.0® visualizations [71].

### 2.3 Ethics approval

The Institutional Review Board of Drexel University provided ethics approval for the Crossroads in Two Colombian Cities Project (no. 2009008067) and the SALURBAL project (ID no. 1612005035). The Ministry of Health and Social protection of Colombia provided the authors a reliance agreement to conduct the research after being presented the study. Both the IRB approval and the reliance agreement approved the use of oral consent for all participants given this was an at-risk population due to migratory status and other intersectional characteristics. Oral consent was documented for all participants in audio before interviews. Written informed consent was not obtained given participants’ weariness to sign documents, disclose personal information and trust institutions including academia. Our ethical approach to the research is based on participatory care ethics [69], which promotes socially responsible action through the re-defining of subjects as active participants instead of vulnerable subjects [72]. Our approach draws from previous work of collective praxis [73, 74]. Adapting to participants’ needs was central to our methodology [60–63].

Because the proposed research involved vulnerable adults and emancipated minors (aged 14–17 years), we conducted extensive consultations with peer-leaders and civil society organizations that provide services to women and girls in the study sites. This work was conducted to identify potential concerns and ethical protections prior to the initiation of our research and to support trust with these individuals through collaboration with organizations on an understanding of the research aims and mechanisms of accountability [72]. We felt that engaging emancipated adolescents was a critically important part of our research in order to ensure a truly inclusive perspective of migrating women and girls’ realities and needs. We determined adolescents were emancipated through community liaisons and peer-leaders who provided care and services to them due to lack of guardians or family members in Colombia. They confirmed so themselves. The research ethics committee specifically waived the need for parental/guardian consent for sampled emancipated minors after consideration of their migratory situation.

No participants were sampled outside of local organizations’ networks and peer-leaders’ referrals. We confirmed emancipation reasons and individual situation during screening. All interview participants received $15 USD incentives for their participation. All participants were informed of their right to leave or terminate the interview at any time.

To ensure protection of both emancipated adolescents and the vulnerable adult participants, research staff involved in data collection activities underwent a 2-month training that covered study aims and objectives, ethical procedures for research with children and adults, referral pathways for participants in need of acute assistance, gender-based violence and child protection concepts and practices, use of audio-equipment, and qualitative methods. All research participants provided informed assent or consent in Spanish. Research staff monitored participants for signs of distress during interviews and reminded participants that they could refuse to participate at any time. Research staff were under close supervision by the P.I. who consulted with researchers after each interview. Participants were prompted to prioritize personal safety and safety of others when doing interviews or discussing the study. Deidentified data was stored on a secure server.

### 2.4 Data integration

Integration of quantitative and qualitative findings is important in concurrent designs. Research question two on how violence impacts HIV risk, prevention, and care in two Colombian cities for VMRW guided the integration. Results of the comparison and integration are exposed in our convergence matrix. This approach enabled us to get a richer understanding of quantitative data [61] and illustrate key aspects of how violence is experienced by VMRW in Colombian municipalities. We merged data [60] following a weaving approach that occurs in the interpretation level and entails presenting quantitative and qualitative findings together as themes [62]. Merging datasets involves bringing the two data sets together for analysis and comparisons after statistical and qualitative analyses are done [62]. We used the interview guide, discussions with our local partner and key informants to assess points of agreement, partial agreement and dissonance between findings [60, 75]. The objective of merging the datasets is to combine the strengths of both while conducting analysis, and assessing how much the data and findings “agree” with each other [60].

## 3. Results

### 3.1 Findings from quantitative analyses

We analyzed data for 84 Colombian municipalities on newly reported HIV cases for 2018. Overall, 13% (n = 11) of municipalities did not report any cases for women. The highest number of reported cases was Bogotá (n = 305); while Cúcuta ranked sixth for the total number of reported cases among women (n = 83); 22 and 5 new cases in foreign-born women were reported for these cities respectively. When looking only at newly reported HIV cases for foreign-born women, 70% (n = 59) of municipalities did not report any cases.

HR varied from no homicides (n = 0 per 100,000) to a count of 79.55 per 100,000. Overall, the newly reported HIV case rate was approximately four times higher for both foreign-born (29.78 per 100,000) and Colombian women (12.36 per 100,000) in municipalities with the highest level of reported HR violence compared to municipalities with the lowest violence level. While differences between quartiles were not statistically significant (ANOVA test p = 0.382 and p = 0.153, respectively), there was a significant positive trend for the HIV case rate across HR quartiles for all women and Colombian women (p = 0.049 and p = 0.042, respectively).

There was no clear trend regarding association of living conditions (p = 0.961), service provision (p = 0.759), and population education (p = 0.572) with levels of violence, as average scores for these variables were similar across quartiles of HR. Even though there were no statistically significant differences, municipalities with the lowest HR have the smallest population (p = 0.354). Population growth shows a marginally significant negative trend with HR increase and there is a higher population growth rate among municipalities with lower levels of HR (p = 0.096). The percent of total migrant population slightly increases with HR quartiles but percent of population who is migrant is not statistically significant (p = 0.471). Regarding population education, the least educated live in municipalities with the highest HR (p = 0.471), however, these differences are not statistically significant (see Table 2).

**Table 2:** Homicide rate distribution according to HIV reported cases for foreign born, national and total women for 84 Colombian municipalities.

Homicide rate (interquartile range) per municipality level	First	Second	Third	Fourth	Overall	
Municipalities (N)	(0–7.43)	(7.91–15.97)	(16.71–26.36)	(26.51–79.55)	(0–79.55)	p value**
	21	21	21	21	84	
Exposure						
Homicide rates (per 100,000 people)	3.04 (3.02)	12.85(2.77)	21.83 (3.18)	46.15 (15.57)	20.97 (17.99)	
Newly reported cases of HIV						
HIV rate in foreign-born women	7.25 (25.81)	16.22 (26.58)	14.58 (20.19)	29.78 (79.69)	16.96 (45.01)	0.382
HIV rate in women nationals	4.94 (7.21)	10.01 (9.39)	15.58 (8.18)	18.91 (6.74)	12.36 (9.47)	0.153
Total HIV rate for women	4.95 (7.47)	10.05 (9.34)	15.52 (8.11)	18.61 (6.83)	12.28 (9.44)	0.163
Municipality level characteristics						
Living conditions	-0.26 (2.35)	-0.09 (1.64)	-0.20 (1.31)	-0.37 (1.45)	-0.23 (1.7)	0.961
Service provision	0.76 (1.79)	1.16 (1.00)	1.14 (0.99)	0.96 (1.49)	1.01 (1.34)	0.759
Population education	55.23 (12.66)	56.19 (11.07)	56.44 (6.88)	52.75 (4.96)	55.15 (9.36)	0.572
Population total	79,796 (74,842.34)	538,883.14 (158,3001.92)	450,357.67 (515,731.49)	324,145.57 (512,329.67)	348,295.6 (873,346.07)	0.354
Population % growth	17.4 (16.22)	17.69 (19.33)	11.67 (12.78)	7.42 (11.60)	13.55 (15.61)	0.096
% of total migrant population	1.61 (1.08)	2.34 (1.67)	2.13 (1.55)	2.57 (3.19)	2.16 (2.03)	0.471
% of female migrant population	0.79 (0.54)	1.15 (0.85)	1.05 (0.78)	1.27 (1.62)	1.06 (1.02)	0.494

* Figures in this table correspond to mean rates and SD for the exposure, dependent variable and across municipalities in each quartile.

** P-values were estimated using ANOVA test with HR as the independent variable, reflecting bivariate associations

### 3.2 Findings from qualitative analyses

We analyzed the interviews following an inductive analysis [71]. VMRW made reference to the relationships between violence, particularly sexual violence, and HIV risks regarding armed conflict dynamics, access to resources and stigma that varied by migration phases (see our convergence table in integration of results for fit of findings with quantitative data). This context includes specific events and practices that expose women to heightened risk given their gender identity and migration status. This conceptualization emerged from findings including two themes: i) *Border crossing and transit through border territories*, which relates to armed actor violence and lack of State accountability in these settings, describing how State corruption, impunity, and lack of access to justice increase HIV risk and exposure for women entering Colombia. Secondly, ii) *Upon re-settlement*, which refers to the increased GBV and lack of access to social resources in Bogotá and Cúcuta that puts VMRW at increased risk for HIV while trying to negotiate housing, jobs, and healthcare, thus limiting social resource access; as well as it refers to HIV and other stigmatized characteristics that increase risk (see Table 3). Below we develop these themes according to agreement of the data and present integrated results.

**Table 3:** Demographics for sampled VMRW (N = 30).

Characteristics		Total (n)	%
Year of migration	2014–2016	3	10%
	2017–2018	18	60%
	2018–2019	18	60%
	2020–2021	3	10%
Main reason for migrating	Economic crisis	15	50%
	Violence/ persecution	5	17%
	Health reasons	6	20%
	Family reunification/ children	4	13%
Age	14–18	6	20%
	19–23	7	23%
	24–28	7	23%
	29–33	3	10%
	34–43	3	10%
	44–48	3	10%
Occupation	sex work/ exploitation	14	47%
	Informal work	8	27%
	Student	2	7%
	Community leader	3	10%
	Unemployed	3	10%
Gender identity	Cisgender	24	80%
	Transgender	6	20%
Race/ethnicity	Mixed race	26	87%
	Black/ Afro Venezuelan	4	13%
Border crossing used	Illegal crossing	16	53%
	Official border pass	14	47%
Knows HIV/AIDS status	Yes	12	40%
	No	14	47%
	Prefers not to report	4	13%
Migration permit	Irregular status	27	90%
	Legal status	3	10%
Children	Yes	20	67%
	No	10	33%
Has experienced direct sexual violence**	Yes	15	50%
	No	6	20%
	Prefers not to report	9	30%
Suffered parental abandonment**	Yes	20	67%
	No	6	13%
	Prefers not to report	4	20%
Family exposed to violence***	Yes	20	67%
	No	7	23%
Teenage mother***	Yes	16	53%
	No	9	30%
	Prefers not to report	5	17%

**Currently or in the past.

*** As currently experienced in re-settlement.

### 3.3. Border crossing and transit through border territories: Armed actor violence control and lack of State accountability

Participants described increased risks and exposure of VMRW to HIV/AIDS at the border between Colombia and Venezuela and border territories they transit through on their way to urban centers. Municipalities closer to the border were consistently identified as a place of danger. A transgender woman explains: “I left because they don’t accept trans women… there’s been a lot of murders… that’s why me and my friends left to come here [to Bogotá].” Around half of participants in this study used illegal border crossings to enter Colombia. Armed groups control border crossings and migration routes, smuggling migrants across illegal paths. In these geographic locations that coincide with migration phases of border crossing and transit, illegal groups and mafias enact raw forms of sexual violence and GBV that perpetrate a culture of lack of access to justice, impunity, corruption, and lack of State accountability. A VMRW recounts:

…if they [armed actors] think you’re pretty, they tell you, you have to sleep with them… we have to have sex with ten men… they make us have sex with ten or eleven men without condoms. Sometimes even Immigration authorities put us in a truck… and throw us on the other side of the border [so they are forced to cross back into Colombia using illegal crossings controlled by armed actors] … I had to be with a guy, and he wanted… to break the condom and I told him no… he got mad and… told me that he was going to leave me… to kill me, eat me… I have to do it, it’s forced… if I don’t do it, I can’t cross. And so, if we try to cross over [through the official border crossing], Immigration authorities… say that we are going to get… deported, that we can spend I don’t know how much time in jail…

Participants described an increased HR risk for women illegally crossing the border, especially for those perceived to have a stigmatized identity like LGBTQ+ individuals, sex workers or younger women. A transgender woman explains: “…I left before they could do anything to me, those people [guerrilla at the border] are treacherous”. However, social violence goes unreported due to fear associated with a lack of a legal migration status and lack of State accountability in territories controlled by armed groups. Both VMRW and KI referred to corruption of State institutions in these locations, oftentimes complicit with armed actors and criminal structures as exposed in the quote. These factors act as deterrents for reporting violence and/or accessing care. Findings expand on HIV risk for VMRW and agreement between levels of violence and HIV risk was found. One of the KI explained how victimization of VMRW happens in border crossings:

An absent State… This is a border territory [Cúcuta and all of Norte de Santander Department], so it is easier for them to smuggle everything they need… drug trafficking, contraband, everything illegal, prostitution. Because war and prostitution are like sisters, no?

Socially enacted GBV is associated to increased risk and exposure to HIV/AIDS when intersecting with other stigmatized identities, like occupation. Almost half of participants engaged in sex work, including 100% of transgender women. Albeit the link between sex work and survival sex is not easily defined in contexts of humanitarian crisis, unmet basic needs, increased risk of violence, illegal border crossing and illegal armed actors’ control, interviews revealed how all of these conditions negatively impact and/or obstruct VMRW’s capacity to negotiate safe sex practices and HIV prevention. Lack of negotiation and stigmatization of women who engage in sex work increases exposure and institutional violence.

VMRW consistently expressed fearing the border and experiencing illegal crossings as traumatic given armed conflict violence and high HR. These factors make VMRW vulnerable to trafficking networks and expose them to heightened HIV risk through kidnapping, torture, sexual and labor exploitation. It also hinders VMRW’s ability to transit and re-settle safely. When armed actors exercise GBV against VMRW it imposes obstacles to their capacity to access protection across migration routes and denounce experiences of violence. A KI explains:

Women entering [Colombia] become interns in these kitchens where they have to cater to all these men doing drug production… Do you know what it’s like to be cooking and have someone grab you from behind, pulling your pants down and raping you right there while you’re forced to keep cooking? In front of everyone else… And try to say no to that, you either get beaten up or… say “throw me in the river, kill me” to avoid getting raped all the time.

Powerlessness to negotiate sex was related to armed actor control at the border, drug production and the need to cross and transit through borderlands to enter Colombia seeking protection, care and social resources. Nevertheless, the experience of migration for half of the women who crossed legally was not significantly different upon resettlement.

### 3.4. Upon re-settlement

#### 3.4.1. Limited access to social resources and HIV risk

**Fig 3:**
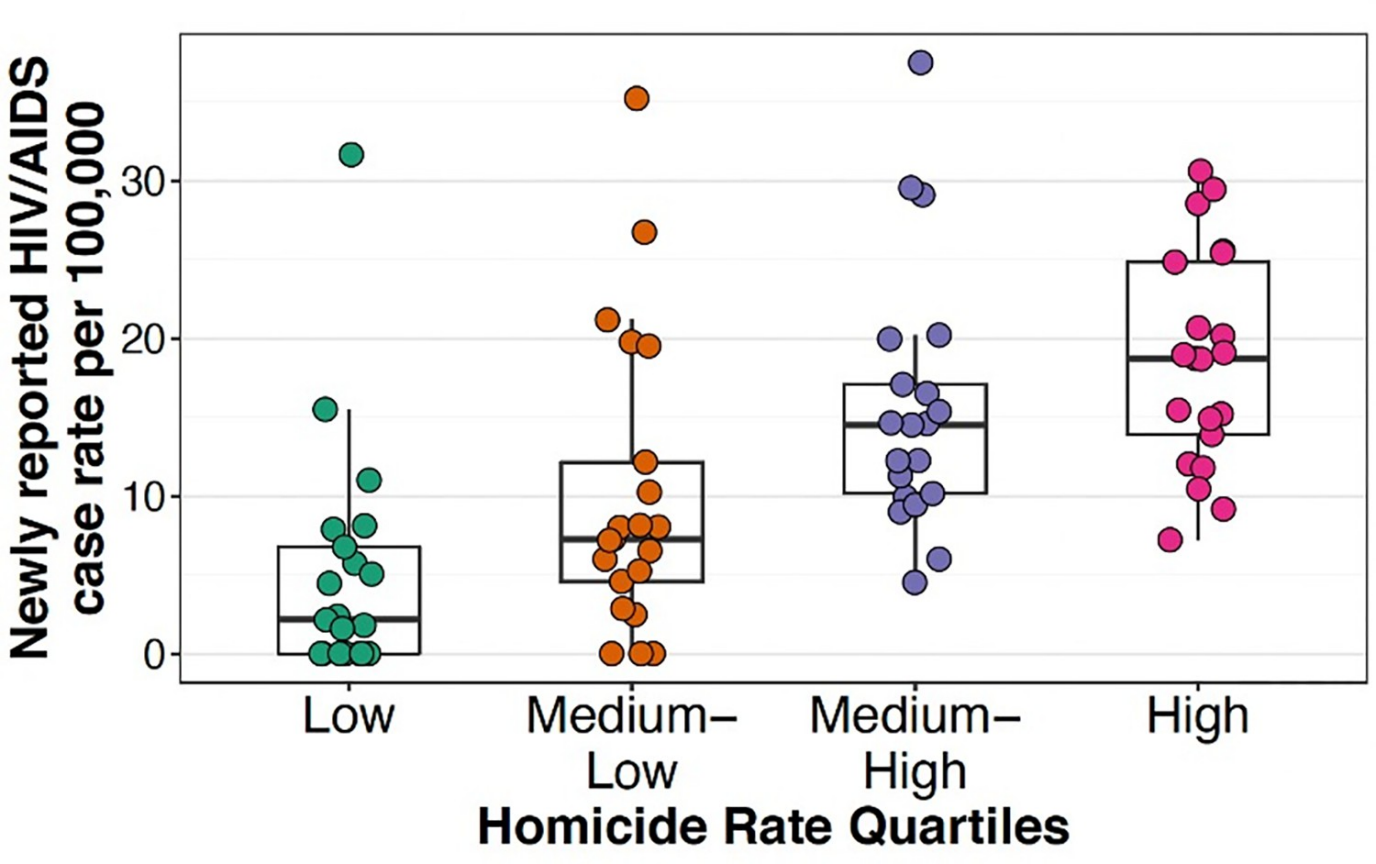
Newly reported HIV/AIDS case rate for women per 100,000 according to homicide rate quartiles (p for trend = 0.049).

We found a statistically significant relation between levels of social violence, conceptualized as HR, and reported HIV/AIDS case rates for 84 Colombian municipalities (see Table 4). We found generally higher newly HIV/AIDS case rates in municipalities in higher quartiles of HR for women. According to negative binomial models each 1-standard deviation increase in the homicide rate (corresponding to 18 homicides per 100,000) was associated with a 34% increase in newly reported case rates (RR = 1.34, 95% CI 1.16 to 1.56). This association was slightly attenuated after adjusting for covariates, but remained statistically significant (RR = 1.25, 95% CI 1.13 to 1.39) (see Table 4). We were unable to run this model separately for Colombian vs foreign-born women, as more than half of the municipalities did not report a single case for the former. The model with the interaction term between HR and foreign-born populations did not yield any evidence of interaction. Fig 3 shows the distribution of newly reported HIV/AIDS case rates by quartile of HR.

**Table 4:** Rate ratios of the association between municipality homicide rates and municipality HIV rates for women.

		Crude	Adjusted**
Variable	Contrast (SD)	RR (95%CI)	RR (95%CI)
Homicide rates*	18.00	1.34 (1.16,1.56)	1.25 (1.13,1.39)

* Homicide rates were standardized to a mean of 0 and a standard deviation of one, the effect represents a 1SD increase in homicide rates

** The model was adjusted for the living conditions and service provision scores, % population 25+ with high school education, population size and population growth for each municipality.

We found agreement between these findings and our qualitative analyses (see Table 5). Upon re-settlement, gender, and migration status impact VMRW’s ability to access social resources (work, housing education). In the case of cisgender women, barriers associate to housing and childcare, whereas in the case of transgender participants, obstacles are related to work opportunities different from prostitution, engagement in webcam services, or transactional sex. Social violence in cities relates to poverty and drug trafficking networks present in neighborhoods where most migrants resettle in Bogotá and Cúcuta and decrease VMRW’s capacity to navigate risk and negotiate protection for them and their families. A KI explains: “neighborhoods have become very dangerous for them [VMRW]… we are talking about contexts that were already very violent”.

**Table 5:** Convergence/fit matrix of key quantitative and qualitative findings.

Findings/ Themes	Quantitative	Qualitative (Meta themes/ themes)	Expansion/ agreement**
HIV case rates is associated with social violence	HR is positively associated with HIV newly reported cases in Colombian municipalities for women.	Violence across migration phases hinders women’s ability to prevent and protect HIV risk. Border crossing and transit through border territories exposes women heightens HIV risk through sexual violence, armed actors’ control and lack of State accountability in border zones.	+ (agreement)
Migration & gender	Rate ratios between municipality homicide rates and municipality HIV rates for women are statistically significant.	Social violence increases women’s risk of HIV by imposing barriers to testing, treatment and prevention upon resettlement. Migratory status, gender and HIV stigma increase violence against VMRW and limit access to social resources.	++ (expansion of quant findings)

**If supporting information was found for quantitative findings in qualitative datasets and it expanded our understanding of the studied phenomena, then a + symbol was used. If contrasting information related to a finding was identified, then a − was used as a symbol. If no information was identified in the data set, then no symbol was placed in the block.

Violence in the resettlement phase is a gendered phenomenon and highly prevalent for VMRW in our sample: half reported having suffered direct violence in their lifetime and about a third preferred not to talk about this. Two thirds of VMRW reported their family being directly exposed to violence upon re-settlement (as exposed in Table 2). A transgender woman describes: “I got stabbed in the lung. I had to spend 15 days at the hospital… someone wanted to rob me”. These experiences expand the focus on how VMRW experience violence in municipalities with high levels of HR. A woman recounted about her rape and trying to go to the police:

…that day I left his house [of the man who raped her] at 3 am and I walked, there was a police station nearby and the policeman asked me what was wrong and then told me I didn’t have a [valid] claim, that because I’m a migrant no one will listen… in that moment I realized nobody cares about me, even sometimes they [the police] are the ones who rape us. During the pandemic they took advantage of women, they did whatever they wanted.

Social violence in Colombian municipalities is a gendered experience conflated with contextual vulnerability. Poverty, labor exploitation, harassment and abuse are everyday situations that interact with migratory status, lack of social support networks, lack of information on system’s functioning and unmet basic needs. Informal work (performed in the street) and sex work also pose challenges. A transgender woman explains: “…they [other people] forced me to fight another woman. She cut my finger… they said I had to earn my spot”. GBV upon resettlement in Bogotá and Cúcuta threatens VMRW’s health when trying to secure living conditions. Sexual abuse is commonly used to resolve where and how to live. A KI explained about Cúcuta:

…they are occupying the mountains already full [of dwellings] so for the plot of land they have to have sex with the owner, and also with the guy who runs the Community Action Board and so if he’s with all of them and they tell you that in nine months all the babies are going to be his it’s because they know they’re not using protection…

Contextual vulnerability due to migratory status and the need to support children and family members, a culturally rooted gendered dynamic for cisgender women, reduces women’s ability to negotiate safe sex practices or access to opportunities upon resettlement, thus increasing HIV risk. We found older women (>24) principally victimized through job exploitation and sexual violence in work and living contexts and adolescents (14–18), who composed around half of our sample, increasingly exposed to GBV through early marriage and sexual abuse. A KI explained: “… adolescents and younger women end up in early marriages with men that double their age, just to sustain themselves, in sexual exploitation situations”. Conversely, transgender women were mostly exploited sexually and through physical and institutional violence perpetrated by clients and police.

#### 3.4.2. Intersectional stigma

Violence impacts VMRW’s HIV risk, prevention, and care in Bogotá and Cúcuta through rigid gender roles, stigmatization of PLWHIV and migratory status. Armed actors’ control of territories extends into cities through drug trafficking networks and crime. They impose extremely high levels of social violence against PLHIV and WLHIV upon re-settlement that creates barriers to access testing and care, especially for VMRW with additional stigmatized identities.

A common strategy to intimidate PLHIV and “purge” communities of “undesired populations” are “social cleansing campaigns” in neighborhoods. The targets of these campaigns comprise sex workers, drug users, LGBTQ+, migrants and PLHIV. The stigma of being a migrant woman who engages in sex work or is involved in “illegal dynamics” (like drug use or distribution) exposes women to these eradication and territory control strategies. These forms of violence often go unreported, as police are identified as complicit with armed groups and identified as victimizers of VMRW. Social cleansing campaigns are especially dangerous for transgender women given perceived violation of gender norms, negative perception of HIV status associated to their identity, migration status, involvement in prostitution and drug use. A transgender woman describes how she got kicked out of a neighborhood by another woman: “Not here f*** Venezuelan… not here you disgusting, f*** piece of infected bitch”

Gender identity exacerbates violence against women given a positive HIV status is socially stigmatized and conceived to conflict with social norms for women. In contexts controlled by armed actors, HIV can be associated with promiscuity due to rigid gender norms and expectations of women’s behavior. Thus, sexually transmitted infections, including HIV/AIDS, represent a risk for personal safety. A KI explains: “Once you become infected of some kind of disease, they [armed actors] take you somewhere and bye. And if not [they do not kill you they say], ‘I see you again and I will kill you’. Let’s say that this is something that marks them.”

Stigmatization of safer practices like using condoms, disclosing positive status, getting tested and being on HIV treatment before and after diagnosis increases intersectional vulnerability for VMRW. Migrants have very little incentives to seek testing and treatment across the care continuum. Armed actors’ control and lack of State accountability are sources of stigmatization, and profiling, which in turn, lead to underreporting of cases in Bogotá and Cúcuta. Ninety percent of our sample reported being HIV negative, but according to our qualitative findings, particularly KI interviews, it is likely that this number does not accurately represent the VMRW population due to strong incentives not to get tested or engaged in care.

## 4. Discussion

Our concurrent mixed method design supports the understanding of how social violence in Colombian municipalities impacts HIV risk for women in general, and for VRMW in particular, describing HIV risk and barriers to care in Colombia.

Quantitative findings show that higher levels of HR were associated with higher rates of newly reported HIV/AIDS cases for women in Colombian municipalities after adjusting for covariates. Qualitative findings describe how HIV risk is displayed across migration phases and relates to gender, migration status and HIV stigma as factors determining women’s ability to negotiate safe sex and navigate border crossing and transit through borderlands in the absence of State presence, as well as access social resources upon resettlement. Our findings describe how particular facets of violence enacted by armed-conflict actors, impunity, corruption and institutional complicity structure present day risks and human rights violations for VMRW, and WLHIV specifically. A novel contribution of our study is that qualitative findings present how HIV risks are differentially experienced by cis- and transgender women across migration phases and point to specific foci where the Colombian State is accountable for human rights violations.

Our qualitative themes illustrate enacted stigma related to how gender stereotyping, profiling and targeted violence against specific intersecting identities imposes barriers for disclosure, testing, treatment, and care for VRMW across migration phases. Through fear and the interplay with lack of information on systems’ functioning, lack of social support and persecution of migrants by State institutions, VMRW experienced increased risks of exposure to HIV when crossing the border and in municipalities under armed actors’ control. Through imposed gender norms, transgender women are at heightened risk of murder and HIV. The facets of violence described here should not be considered exhaustive. Intergenerational trauma, adverse child experiences, direct trauma and mental health consequences of violence are only partially constitutive of the structural violence that imposes emotional, mental and physical distress on women and girls in LAC [76].

Our analyses concur with evidence in the region on the relationship between social violence as related to poverty [39], inequality, impunity, corruption, and/or the presence of organized crime [37]. Armed actors territory control represents an increase in violence and impunity of crimes, a strong deterrent to access healthcare services and justice. This makes both violence and HIV cases subject to underreporting. In borderlands controlled by armed groups, violence and HIV risk for cisgender women relates to the drug production industry. Women are targeted given socially sanctioned roles and expected to fulfil sexual and labor expectations that impact their ability to negotiate safe sex and protection. For transgender women, armed actors’ presence increases the risk of physical violence and expulsion of territories. Findings are consistent with existing literature in LAC stating that social violence increases [41] with the prevalence of organized crime [37], drug trafficking, criminal economies [27], and institutional weakness [43]. All women reported fearing and systematically experiencing traumatic events at border crossing and transit [77]. These accounts link experienced violence to gender and victimization of VMRW by armed groups. Practices of forced recruitment, sexual violence, extorsion, kidnapping and physical violence increase HIV risk for VMRW in these migration phases. The intersection of stigmatized identities [78] (i.e. engaging in sex work, being younger or LGBTQ+) increase the risk of suffering violence and being exposed to HIV.

We found greater exposure to HIV risk through different forms of sexual violence during border crossing, transit, and resettlement. The *border crossing theme* presents violence to be more prevalent among women in Cúcuta, women engaging in sex work and younger women. Resettlement violence and lack of access to social resources was equally prevalent in both study sites. Barriers to meet basic needs, job security, educational attainment, living conditions and poor access to healthcare services are also related to increased risk in cities given women’s need to secure safe living conditions for both cis- and transgender participants. Social class and gender identity may act as modifiers of health inequalities, but these are exacerbated for populations that migrate from and to poorer areas [79]. Thus, an intersectional stigma approach [78] to VMRW’s health and the health needs of transient populations is necessary.

Convergence of findings allows us to explain how the control of territories by armed groups across migration phases intertwines with HIV risk and violence for VMRW. Violence hinders the protection of human rights and access to justice given migrants’ lack of institutional knowledge and fear of deportation, imposing barriers to HIV testing, treatment, and care upon resettlement. Institutional actors like police and immigration authorities reinforce stigma and barriers through discrimination and xenophobia. Findings might explain why there is a lack of HIV case reports in most Colombian municipalities for foreign-born women. While this may be due to lower case rates, there is also the possibility of underreporting of new cases because of perceived and actual barriers, as pointed out in the *upon re-settlement* theme. Lack of reporting might also relate to gender stereotyping and intersectional stigmatization, as well as to social violence enacted against PLHIV by armed actors permeating urban spaces.

Research in other contexts relates violence and hegemonic gender roles to a context of violence against WLHIV [80], where gender acts as a system of social stratification and control that influences access to power, status and material resources [81]. In Colombia, culturally sanctioned gender roles and stereotypes have been connected with exacerbated violence against VMRW [77] -especially for trans women-[52] and entail increased vulnerability to HIV [82]. The humanitarian crisis has also propagated stereotypes of VMRW as being HIV positive and experiencing other negative health outcomes [82]. Gender roles increase violence against people perceived as violating such norms, like sex workers or LGBTQ+ individuals. Risk and exposure across migration phases cannot be fully understood independently of these social dynamics and how they intersectionally affect women’s health [78], HIV prevention and care, exposing how gender interacts with other identities to embody risks for women [4].

We describe how social violence, gender and migratory status, occupation and poverty increase the risk of HIV through direct sexual violence, harassment, and abuse across migration phases. These findings are consistent with literature from other contexts linking migration and gender to women’s risk of exposure and infection of HIV, shaping risks and embodied experiences of suffering ACEs, child abuse, neglect, forced prostitution, torture and selected killings [80]^.^ These factors increase gender inequality and contribute to social vulnerability [4]. Our findings are also consistent with the robust body of literature on the GBV-HIV dynamic [83].

We understand HIV risk in a continuum along migration phases that informs where resources should be allocated and would be most useful to prevent HIV risk and violence against women. Institutional accountability -including that of the Colombian State- and control of border territories, as well as State presence and access to services would be beneficial to guarantee protection of human rights and prevention against violence for VMRW in border crossing and transit phases. This is an obligation of the government under the Constitution, as health is a right that should be guaranteed. Upon re-settlement, tending to safe living conditions related to housing, education and job opportunities is paramount to protect VMRW and decrease HIV risk [41]. Improving access to social resources would not only contribute to socioeconomic security but to access insurance through work. Strengthening social and information systems would also prove beneficial to connect migrants to social support services. These are some main recommendations to improve human rights protections and hold the government accountable for institutional violence.

As Colombia moves forward to implement a ten-year plan to provide migrants with a legal migratory status [55], social violence should be discussed as a barrier to access social resources and a factor impacting VMRW’s health. Our study has implications for understanding and addressing HIV/AIDS risk across migration phases within the larger context of a humanitarian crisis in the region, where most Venezuelan migrants are re-settling in urban centers [26]. Our study sheds light on how to support VMRW and PLHIV who face armed actors’ control and stigmatization, illustrating how social violence imposes barriers to access testing and treatment across the care continuum. Research in LAC is still scarce on how social violence impacts migrant’s health and further implementation of participatory frameworks that include women’s voices is warranted. It is recommendable that international agencies and foreign governments support and fund continuous processes in the middle- and long-term to maximize the impact of HIV and violence prevention and protection strategies, as well as to support the Colombian government’s obligations under human rights law.

## 5. Limitations

Underreporting of homicide rates and mortality data is an issue in LAC, especially for poorer regions and areas [84]. Given that we use HR as a proxy for social violence, a concern on reports of rates for poorer municipalities is warranted. Given our qualitative findings, it is possible that HIV cases are also underreported, particularly for WLHIV. Therefore, we believe that the estimates of associations we obtained are a conservative estimate, as we hypothesize that the underreporting of cases would be higher in areas with higher levels of social violence and may be particularly more acute for cases among foreign-born women. This underreporting of cases has limited our ability to examine case rates among Colombian and foreign-born women separately.

There is a possible underestimation of the population denominator in the Venezuelan-born group given high-mobility dynamics [26], lack of legal status and perceived stigmatization of State’s institutions by this population. Nevertheless, we believe our qualitative findings to be strong enough to provide an explanation of both the underreporting of cases and why social violence impacts HIV rates for women, particularly for VMRW. Given the ecological nature of our quantitative analysis, results should not be interpreted individually (i.e., that the individual experience of violence is associated with higher HIV risk). While our objective was not to estimate causal effects, there is still a possibility of residual or unmeasured confounding in our associations. This may lead to either over or underestimation of the true association. For example, if municipalities with higher levels of income inequality tend to have both higher violence and number of HIV cases, part of our observed association could be explained by these differences in income inequality. Despite adjusting for municipal social environment scores and population education level, the lack of available data at the municipality level limited our ability to adjust for other economic and social factors such as income inequality. There is also potential for reverse causation as we do not know the exact timing for infection or homicides. Lastly, we are unable to provide accurate estimates for incidence of HIV in the Venezuelan migrant population.

Our qualitative findings are informed by our partnership with local organizations and may be skewed towards the population they work with. Findings in Bogotá and Cúcuta cannot be interpreted as common across Colombian or LAC cities. We believe our methodology mitigates social desirability bias, promotes honesty and is informative of VMRW’s experiences given our participatory approach, and long-term collaboration with VMRWG, local partners, and community educators.

## 6. Conclusions

We achieved our first aim by understanding how violence is associated with newly reported HIV/AIDS case rates for women in Colombian municipalities. We found that, at the municipal level, higher HR are associated with higher rates of newly reported HIV/AIDS cases for women. We achieved our second aim by describing how social violence impacts HIV risk, treatment, and prevention specifically for VMRW in two Colombian cities through stigmatization, armed actors’ control, presence of criminal economies and social violence.

Our study informs HIV policies that aim to tackle growing HIV case rates and interventions on social violence against migrants and refugees in two Colombian urban centers. The factors identified as impacting HIV risk for women call for a unified intersectoral and intersectional response. We present a graphic documentation of the severe and pervasive violence against VMRW in Colombia, the helplessness created by the lack of legal status and lack of structures that provide any oversight over armed actors, corruption, impunity, and lack of accountability of the Colombian State.

We contribute to the scarcity of research in Colombia and the region that presents violence as a contextual modifier of risk of HIV/AIDS for women across migration phases. Innovative solutions should go beyond any single nation and require a coordinated plan to ensure that people migrating across borders, particularly women and girls, have their fundamental human rights protected. Health systems in countries receiving migrants from Venezuela and elsewhere should be strengthened so that healthcare needs of migrants and refugees can be met without negative consequences for local HIV programs and receiving populations. Intersectoral work, international monitoring and surveillance of violence against migrants and especially women, a gender approach to health systems that includes an integration of sexual and reproductive policies with mental health care and social resources are specific strategies for healthcare systems in the region to tackle challenges associated with incoming migration flows related to this or other humanitarian crises.
